# *Vaccaria segetalis*: A Review of Ethnomedicinal, Phytochemical, Pharmacological, and Toxicological Findings

**DOI:** 10.3389/fchem.2021.666280

**Published:** 2021-04-29

**Authors:** Meng Tian, Yuwen Huang, Xin Wang, Maosheng Cao, Zijiao Zhao, Tong Chen, Chenfeng Yuan, Nan Wang, Boqi Zhang, Chunjin Li, Xu Zhou

**Affiliations:** College of Animal Sciences, Jilin University, Changchun, China

**Keywords:** *Vaccaria segetalis*, prolactin activity, estrogen-like activity, anti-tumor activity, anti-oxidant activation

## Abstract

*Vaccaria segetalis* is a dry mature seed of Vaccaria hispanica (Mill.) Rauschert, which belongs to the genus *V. segetalis* (Neck.) Garcke. There are multiple medicinal parts of *V. segetalis*, according to the records, including roots, stems, leaves, flowers, and seeds, which should be used together. Currently, *V. segetalis* is most frequently used in the treatment of menstruation, dysmenorrhea, breast milk stoppages, and chylorrhea. Numerous studies present historical evidence of the use of *V. segetalis* to treat several diseases and describe its beneficial effects including prolactin- (PRL-) like, estrogen-like, antitumor, antiangiogenesis, and antioxidant activity. We summarized the period from January 1980 to December 2019 regarding *V. segetalis*. This review paper indicates that *V. segetalis* has promising clinical applications. The main active ingredients of the plant have been elucidated in recent years. We summarized the previously and newly discovered pharmacological effects of *V. segetalis* in addition to its active ingredients, ethnopharmacological uses, and toxicological properties, and provided a focus for future research.

## Introduction

*Vaccaria segetalis* (Neck.) Garcke is an annual herb and is widely distributed worldwide throughout the cold temperature zone. The seeds of *V. segetalis*, which are known as Wang Bu Liu Xing, have been used in traditional Chinese medicine (TCM) to treat amenorrhea, dysmenorrhea, lactation failures, and carbuncles (Sang et al., 2003). *V. segetalis* has been used in China for 2000 years as documented in the oldest materia medica *Shen Nong Ben Cao Jing*. In recent years, being its clinical application more extensive, and many new clinical applications, such as the treatment of shingles (Min-ying, 2005), gallstones (Zhi-hong and Cai-ying, 1989), hypertension (Liu, 2018), and rhinitis (Liang and Xiu-jun, 2013), have been identified. *V. segetalis* is composed of several chemical components. Saponins (Ma et al., 2008), flavonoids (Wang et al., 2011), polysaccharides (Qing et al., 2014), and cyclic peptides (Sang et al., 2002) are believed to be the principal active constituents of *V. segetalis*. The seeds also contain components such as coumarins, lipids, fatty acids, and metallic elements (Jin-Ling et al., 2014). Recently, an increasing number of studies have suggested that *V. segetalis* extract has various bioactivities, such as prolactin- (PRL-) like, estrogen-like, antitumor, antiangiogenesis, and antioxidant activity, and it is also shown to dilate blood vessels and relieve osteoporosis (Li-Fan and Liang, 2007). This article also summarizes toxicological research regarding *V. segetalis*.

Complementary/alternative medicine has developed rapidly in recent years, and its use has gradually expanded globally. The use of Chinese herbal medicine as a part of complementary/alternative medicine has been the focus of many scholars. Although a few literature studies have described *V. segetalis* to date, no article has systematically summarized and evaluated the research results. We conducted this review to summarize the existing literature studies and to focus on analyses of the chemical constituents and pharmacological activities of *V. segetalis*. Our goal is to permit other researchers to more easily review existing research and provide new directions for future research.

## Ethnopharmacological Uses

### Preparation of *V. segetalis*

Dry mature seeds from *V. segetalis* were prepared by using a multistep process. *V. segetalis* has different traits in different preparation stages (Tian-Yi, 2011). A specific preparation method is described below. In the first stage, after the fruits mature in summer, the plants are cut and dried. Then, the seeds are beat and dried. During this time, the seed is a black sphere with a diameter of approximately 2 mm. In the second stage, the seeds are fried until most seeds burst into white flowers. In the final stage, the seeds that burst into white flowers are ground into powder.

### Methods of Administration

There are three administration methods for different preparation stages of *V. segetalis* because of its different traits. In the first type, seeds can be applied directly by pressing acupoints. In the second type, the seeds are fried until the white flowers burst, and the seeds are decocted in water. In the third type, the ground powder is applied directly to the affected area.

### Indication

The seeds of *V. segetalis* are often used as black spheres in acupressure methods. Based on the TCM theory, acupoints are the specifically chosen sites for physical stimulation (Rong et al., 2011). A few studies have revealed that stimulating different acupoints on the body surface could provide various therapeutic benefits (Li et al., 2015). Previous research illustrated that pressing acupoints with seeds can improve hypertension. In one study, Liu (2018) selected the auricular points that were related to blood pressure, pressed one seed at each acupoint, and kneaded each acupoint for 3–5 min. This treatment was performed thrice a day, and the contralateral acupoints were alternatively pressed every 3 days. After 2 weeks of treatment, the hypertension symptoms of a patient had resolved (Liu, 2018). Gallstones can also be treated in this manner. In another study, the researchers used the seeds of *V. segetalis* to press the gallbladder, liver, duodenum, and sympathetic points on both sides of the ears of patients dozens of times and found that the gallbladder contracted significantly. Consequently, small stones and silt-like stones in the gallbladder were discharged into the intestinal lumen with bile, and then excreted from the body (Zhi-hong and Cai-ying, 1989). In addition, this method was used to treat rhinitis. A research team also used the seeds to press the ear points that were related to the nose 3–5 times a day, for 1–3 min each time. Ear points were changed every 3 days, switching between the two ears, and the course of treatment was 10 days (Liang and Xiu-jun, 2013). *V. segetalis* as a white flower after frying is often used in water decoctions. *V. segetalis* has also been used in combination with other Chinese herbal medicine to treat gonorrhea and promote the milk production. According to *The Precious Mirror of Hygiene* (AD 1343), ancient Chinese herbalists used *V. segetalis* to boost the milk production and milk lactation. The traditional Chinese herbal medicine prescription consists of equal amount of the spike of *Dianthus superbus* L., *Ophiopogon japonicas* (L.f.) Ker-Gawl (inner column removal), *V. segetalis*, fossil fragments (ancient mammals such as elephants, rhinos, three-toed horses, cattle, and deer), and the scale of *Manis pentadactyla* Linnaeus to the total of 50 g. The components are mixed and ground into a powder. In total, 5 g of the mixture are consumed with hot wine thrice a day. Then, 5 g of the mixture are added to a soup consisting of pork knuckle and *Akebia quinata* (Houtt.) Decne. and consumed thrice a day. Finally, the remaining mixture is rubbed on the left and right breasts 30 times with a wooden comb after consuming the aforementioned soup thrice a day (Tian-Yi, 2011). In recent years, a new application method has been to grind the white flower into a powder for an external application on the affected area. This was proven to be effective for treating shingles (Min-ying, 2005).

In summary, the applications of *V. segetalis* have been gradually expanded and improved with the accumulated research data. *V. segetalis* can be used externally or internally. It has been used for a long time based on the physical properties of the seeds to stimulate acupuncture points to treat diseases and their mixing in water decoctions with the other Chinese herbs. The newest application method is a direct application of the powder to the wound surface to treat shingles. We speculate that these activities are related to the active ingredients in seeds. We believe that the in-depth studies of *V. segetalis* will increase its clinical applications.

## Primary Active Ingredients in *V. SEGETALIS*

*Vaccaria segetalis* is mainly composed of saponins, cyclic peptides, flavonoids, and polysaccharides, and the other components include volatile oils, coumarins, lipids, and fatty acids. Several studies have investigated these active substance constituents.

### Saponins

Several types of saponins are present in the seeds of *V. segetalis*. These saponins primarily include quillaic acid compounds, which account for ~65% of the total (Table 1). The other saponins include gypsogenin bisdesmosides (~15%), gypsogenic acid monodesmosides (~10%), and vaccaric acid bisdesmosides (~10%). The structures of the known saponins are summarized in Table 1. Triterpenoid saponins are the main components of *V. segetalis*. They are oleanol-type pentacyclic triterpenoids, which are also known as β-aromatic alkanes. These compounds are widely distributed in the plant kingdom. Triterpenoid saponins mostly have antioxidant (Guan et al., 2013), antitumor (Bozak et al., 1990; Haralampidis et al., 2002; Choi et al., 2005), and anti-inflammatory effects (Bernard et al., 2001; Banno et al., 2005; Guan et al., 2013). They also protect the cardiovascular system through their antihypertensive, anti-atherosclerotic, and vasodilatory effects (Rosalía et al., 2004). The main pharmacological activities of *Panax ginseng* C. A. Meyer, *Glycyrrhiza uralensis* Fisch., and *Bupleurum L*, which are widely used in daily life, are derived from their saponin components.

**Table 1:** Saponins present in the seeds of *Vaccaria segetalis*.

**Saponins**	**Formulae**	**Structure**	**Pharmacological activities**	**References**
**Segetosides**				
Segetoside B	C_69_H_106_O_33_	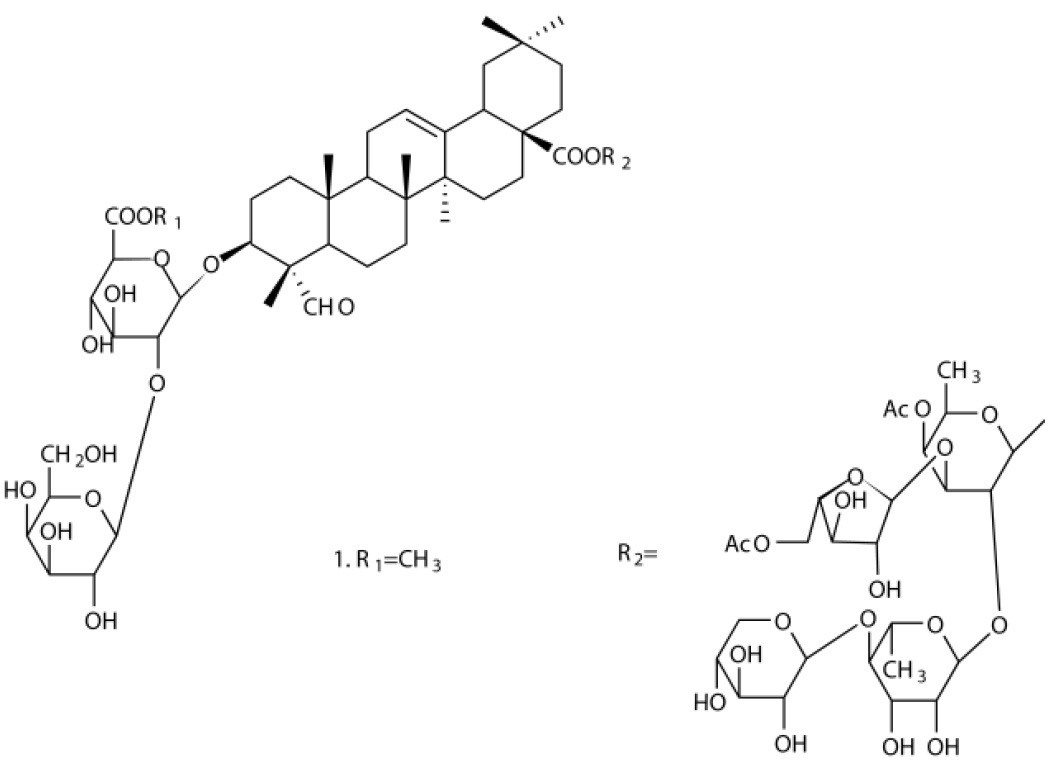	Inhibition of luteal cell	Sang et al., 2002
Segetoside C	C_56_H_88_O_32_	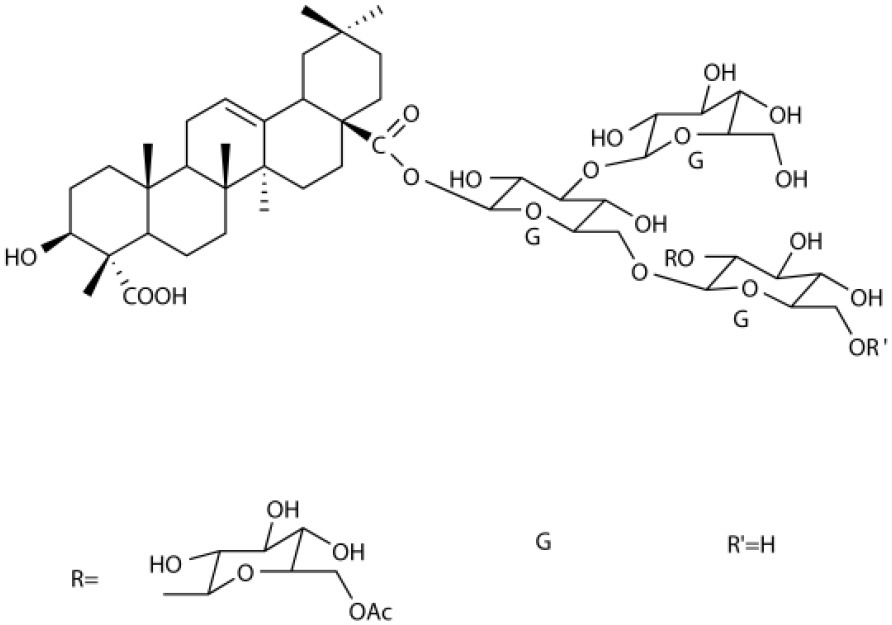	Unknown	Sang et al., 1999
Segetoside(D–E)	Common structure	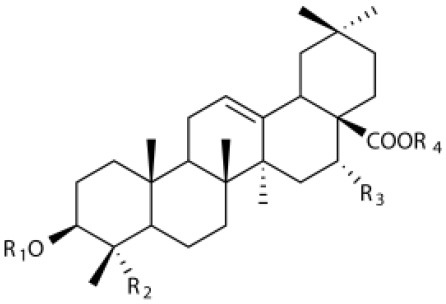		
Segetoside D	C_69_H_106_O_34_	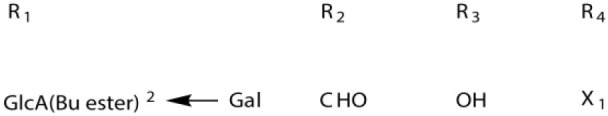	Anti-cancer	Güçlü-Ustündag and Mazza, 2007
Segetoside E	C_72_H_112_O_34_	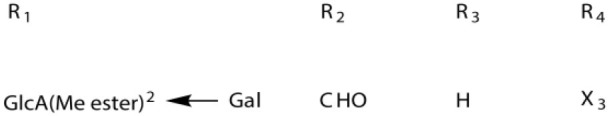	Unknown	Sang et al., 1999
Segetoside(F–I)	Common structure	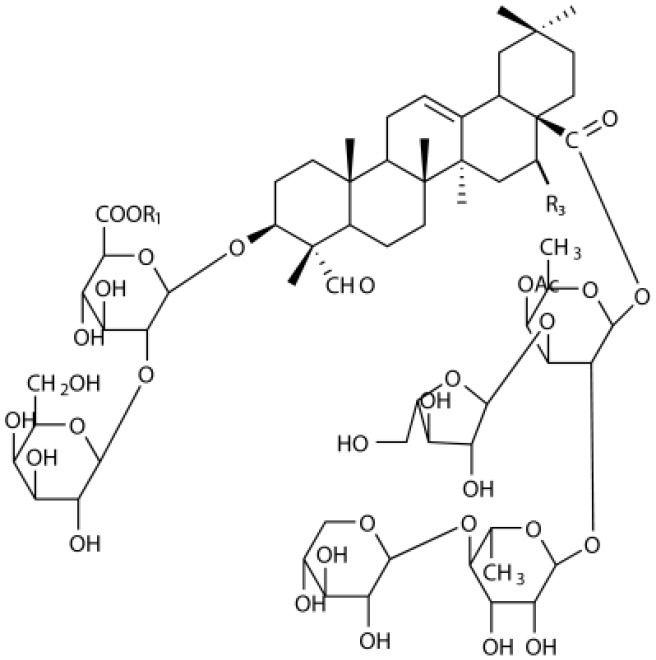		
Segetoside F	C_67_H_104_O_32_	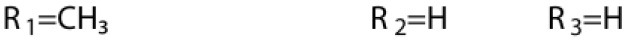	Inhibition of luteal cell	Sang et al., 2000d
Segetoside G	C_70_H_110_O_32_	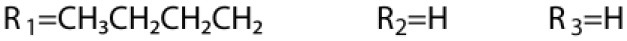	Unknown	Sang et al., 2000a
Segetoside H	C_68_H_104_O_33_	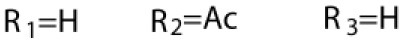	Unknown	Sang et al., 2000a
Segetoside I	C_68_H_104_O_34_	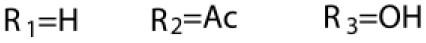	Anti-tumor activity; Activation of apoptotic	Sang et al., 2000b
Segetoside K	C_54_H_86_O_26_	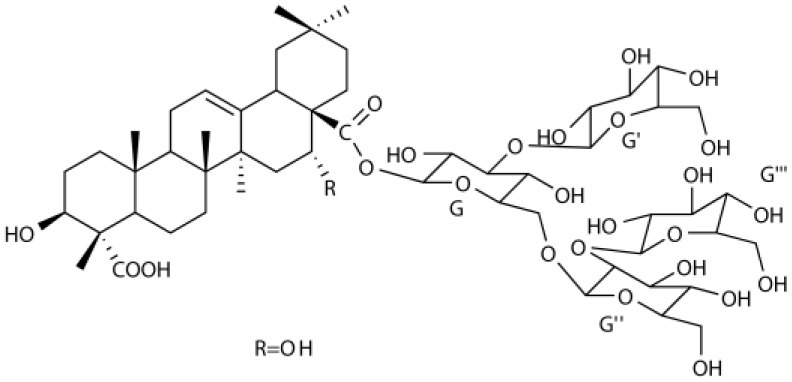	Unknown	Sang et al., 2000a
Segetoside L	C_60_H_98_O_28_	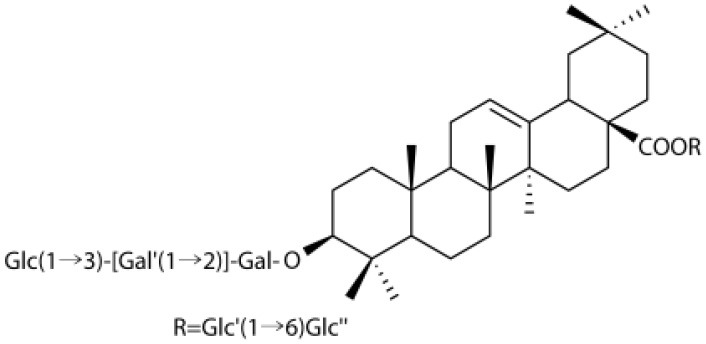	Unknown	Minliangzou and Ainalao, 1999
**Vaccarosides**				
Vaccaroside (A–C)	Common structure	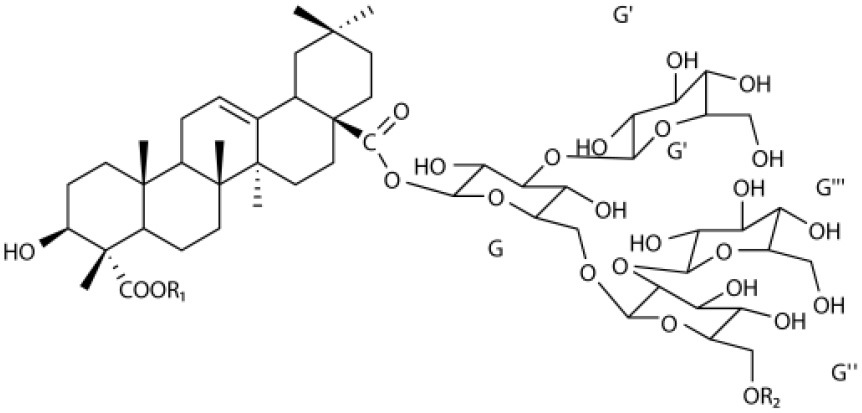		
Vaccaroside A	C_54_H_86_O_25_	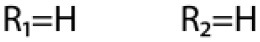	Unknown	Sang et al., 1999
Vaccaroside B	C_60_H_94_O_29_	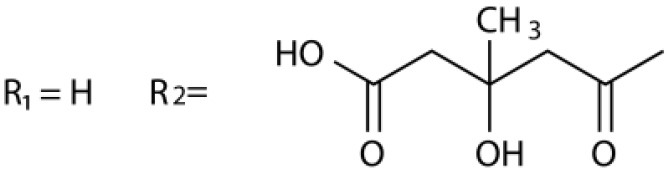	Unknown	Ma et al., 2008
Vaccaroside C	C_54_H_86_O_25_	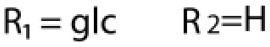	Unknown	Koike et al., 1998
Vaccaroside D	C_54_H_86_O_25_	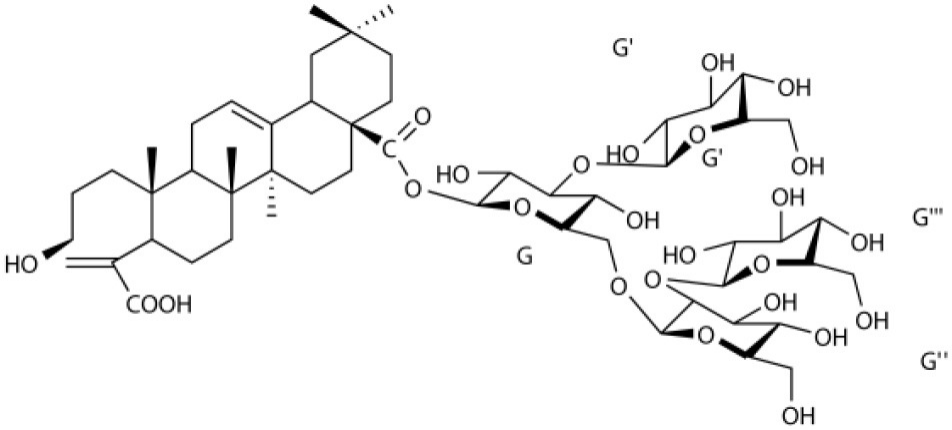	Unknown	Koike et al., 1998
Vaccaroside (E–H)	Common structure	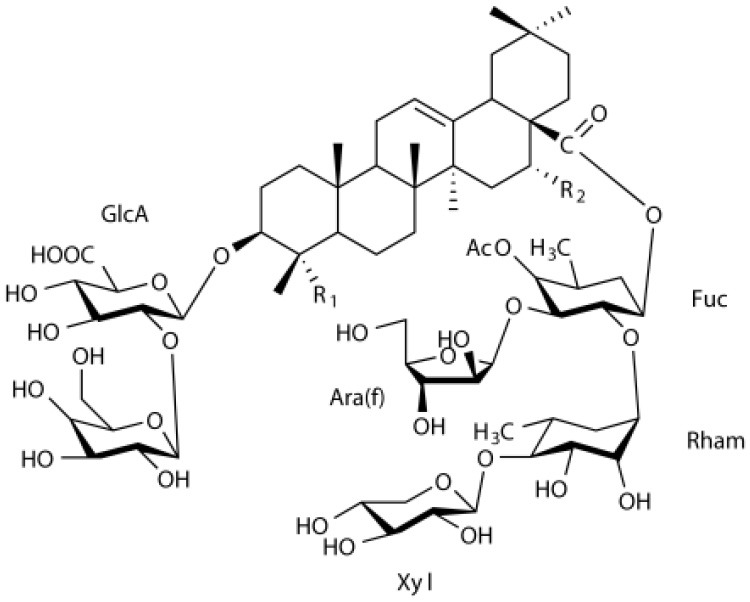		
Vaccaroside E	C_66_H_102_O_33_	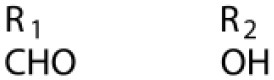	Unknown	Jia et al., 1998
Vaccaroside F	C_65_H_102_O_33_	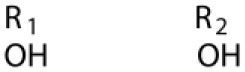	Unknown	Jia et al., 1998
Vaccaroside G	C_66_H_102_O_32_	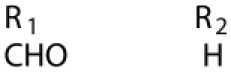	Unknown	Jia et al., 1998
Vaccaroside H	C_65_H_102_O_32_	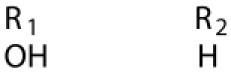	Unknown	Jia et al., 1998
Vaccaroside I	C_71_H_112_O_37_	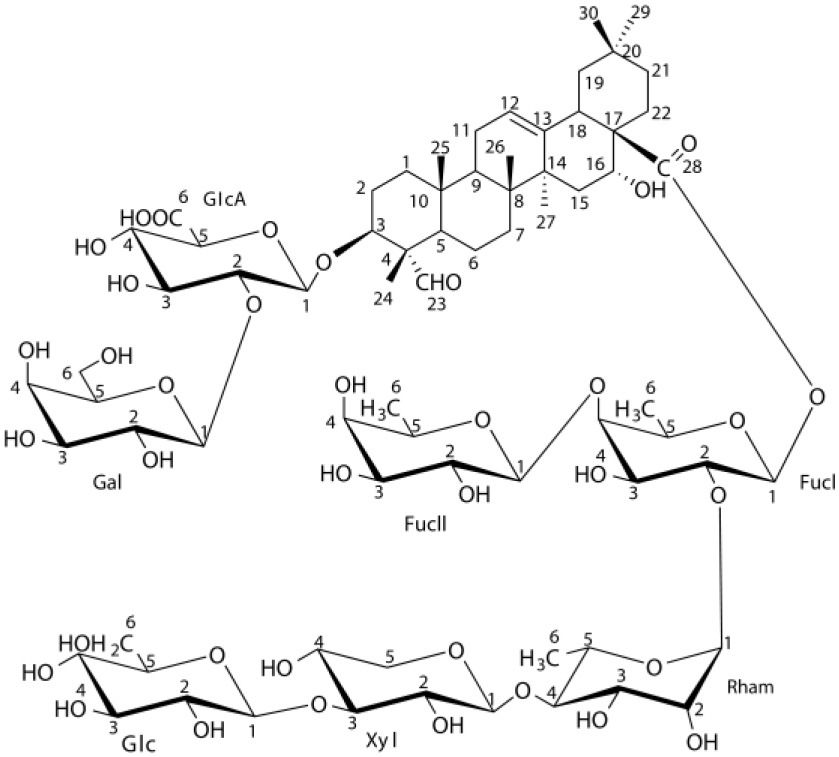	Unknown	Ma et al., 2008

### Cyclic Peptides

Cyclic peptides have been found in several medicinal species such as *Pseudostellaria heterophylla* Miq., *Lycium chinense* Mill., and *Psammosilene tunicoides*, and exhibit a wide range of structure-dependent bioactivities (Tan and Zhou, 2006). Cyclic peptides are important components of *V. segetalis* (Dahiya and Dahiya, 2021). With the development and integration of modern medicine and traditional complementary medicine, the roles of cyclic peptides have been further confirmed. To date, eight cyclic peptides have been isolated from the seeds of *V. segetalis* (Table 2). Cyclic peptide molecules have antitumor activity (Feng et al., 2012), and they regulate uterine contraction, which implies estrogen-like activity for segetalins A, B, G, and H (Itokawa et al., 1995; Yun et al., 1997). In addition, a few studies illustrated that segetalins A, D, F, and G have vasodilatory activity against norepinephrine-induced aortic contraction in rats (Morita et al., 1997a). The pharmacological effects of cyclic peptides provide new options for the treatment of diseases.

**Table 2:** Cyclic peptides present in the seeds of *V. segetalis*.

**Cyclic peptide**	**Formulae**	**Structure**	**Pharmacological activities**	**References**
Segetalins A	C_31_H_43_N_7_O_6_	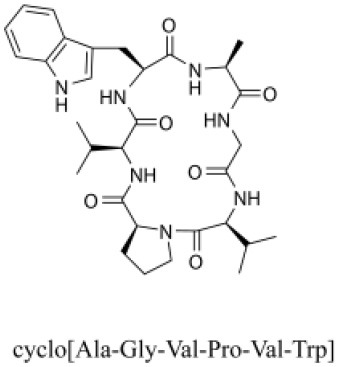	Vasorelaxant activity; Estrogen-like activity	Morita et al., 2006
Segetalins B	C_24_H_32_N_6_O_5_	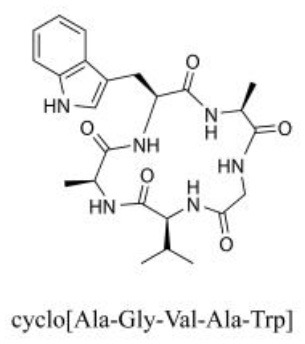	Estrogen-like activity	Morita et al., 1997b
Segetalins C	C_40_H_51_N_9_O_7_	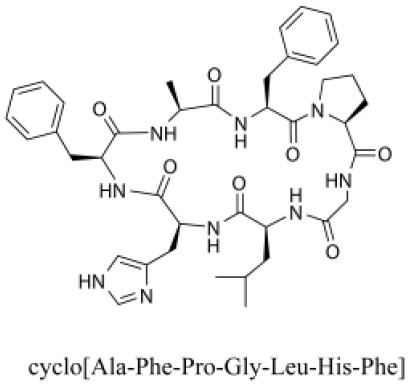	Antimicrobial activity	Dahiya and Kaur, 2008
Segetalins D	C_37_H_49_N_7_O_8_	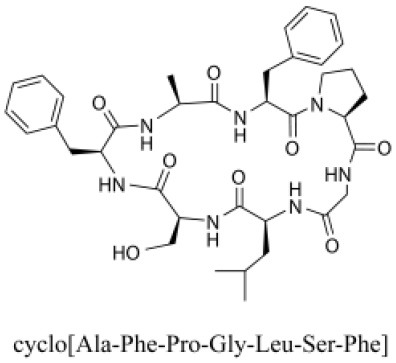	Estrogen-like activity	Morita et al., 1997a
Segetalins E	C_43_H_56_N_8_O_8_	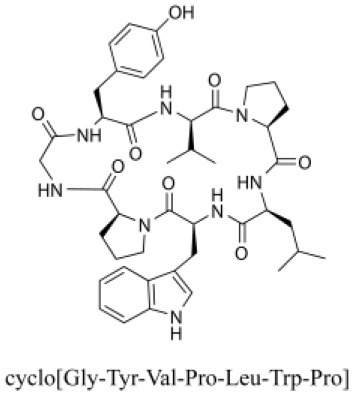	Unknown	Sang et al., 2000d
Segetalins F	C_44_H_62_N_10_O_14_	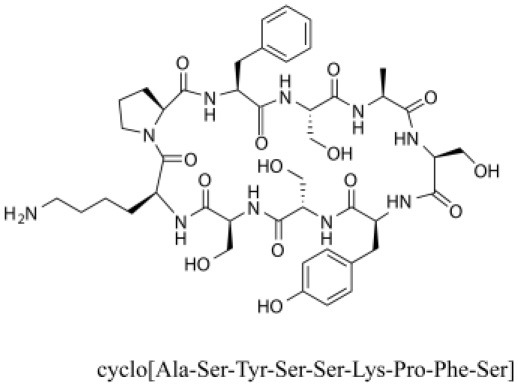	Vasorelaxant activity	Morita et al., 2006
Segetalins G	C_25_H_38_N_6_O_6_	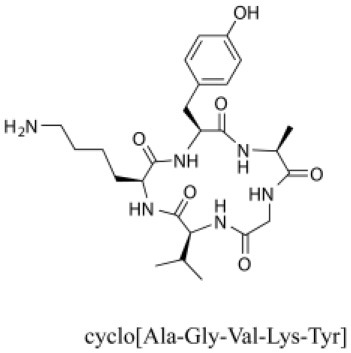	Estrogen-like activity; Diastolic activity	Yun et al., 1997
Segetalins H	C_29_H_38_N_8_O_8_	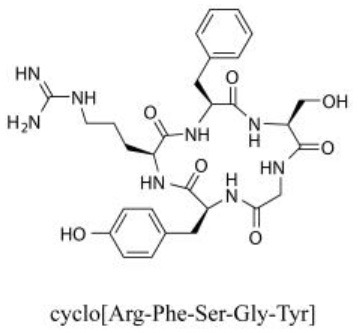	Estrogen-like activity; Vasodilatory activity	Yun et al., 1997

### Flavonoids

**Figure 1:**
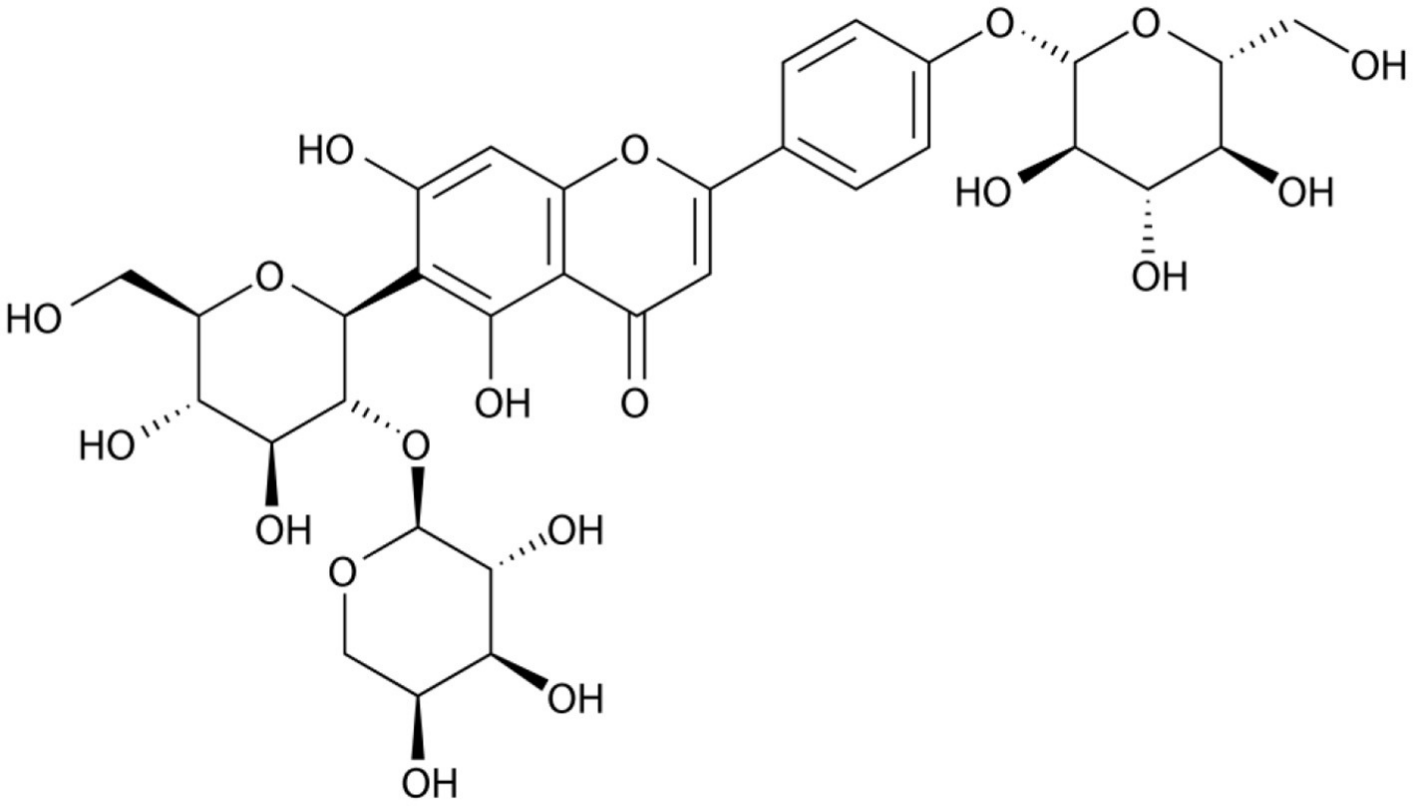
Structure of vaccarin.

Flavonoids comprise a broad class of ketone containing compounds that exist widely in nature. Flavonoids are widely distributed in plants in the form of glycosides, which are called flavonoid glycosides. In particular, *Sophora japonica, Scutellaria baicalensis, Pueraria lobata*, and *Ginkgo biloba* are rich in flavonoids. The structure of vaccarin, a flavone isolated from the seeds of *V. segetalis*, is presented in Figure 1 (Sang et al., 2000c). Flavonoids have many effects, such as protective effects on endothelial cells (Kozlowska and Szostak-Wegierek, 2014), hypoglycemic effects (Chen et al., 2017), antiviral effects (Zakaryan et al., 2017), and the ability to enhance lactation capacity (Tong et al., 2013).

### Polysaccharides

Polysaccharides are formed by the polymerization of more than 10 monosaccharide molecules *via* glycosidic bonds. They have high molecular weights, and they usually consist of several hundreds to thousands of monosaccharide molecules. Polysaccharides and their derivatives, such as lentinan, *Astragalus* polysaccharide, *Polyporus* polysaccharide, *Lycium barbarum* polysaccharide, and *Ganoderma lucidum* polysaccharide, are bioactive substances in TCM. They have antitumor (Mao et al., 2016; Tang et al., 2016), antioxidant (Hui et al., 2019), and anti-inflammatory effects (Wang et al., 2019), and promote cell proliferation (Zheng et al., 2014). Polysaccharides have been isolated from the seeds of *V. segetalis* in recent years (Zhou et al., 2015). Various active ingredients in *V. segetalis* provide a theoretical and realistic basis for the study of its pharmacological effects.

### Other Components of *V. segetalis*

*V. segetalis* also contains coumarins, lipids, fatty acids, and other ingredients, along with adenosine and adenine. The main components of *V. segetalis* volatile oils are oleic acid amide, n-octadecane, myristylamide, and n-pentadecane (Yun et al., 1998). In addition to the currently known major active ingredients, several unknown ingredients of the plant require further investigation to be identified.

## Pharmacological Activity of *V. SEGETALIS*

Recently, *V. segetalis* has mainly been used in folk medicine based on its anti-inflammatory, antioxidant, anti-angiogenic, and antitumor effects, in addition to its ability to promote vasodilation, muscle contraction, and lactation. The active ingredients responsible for these effects are polysaccharides, saponins, flavonoids, and cyclic peptides (Peng et al., 2014). We will now specifically introduce its pharmacological activities.

### Anti-inflammatory Activity

According to *Shen Nong Ben Cao Jing, V. segetalis* has been used to treat urinary symptoms such as blood strangury for 2000 years. Gonorrhea in Chinese medicine refers to diseases such as urinary tract infection, prostatitis, and seminal vesicle inflammation. The traditional Chinese herbal medicine prescription is as follows: 30 g of the seeds of *V. segetalis* and 6 g each of the roots of *Angelica sinensis* Diels, *Chinese teasel, Paeonia lactiflora* Pall., and *Salvia miltiorrhiza* are orally administered as two doses *via* decoction (Zhu-mo, 2005). Based on an extensive application of *V. segetalis* in the treatment of inflammatory diseases, we searched and collated the articles published in the past 30 years to explore the intrinsic mechanism of *V. segetalis* in the treatment of inflammatory diseases.

Hypaphorine from the different sources exhibits anti-inflammatory properties (Silva et al., 2012). *V. segetalis* extract, along with its hypaphorine, displayed anti-inflammatory activity both *in vitro* and *in vivo* (Aswad et al., 2018). In this study, mice were intraperitoneally injected with 200 mg/kg of the 6.019% of *Vaccaria* hydroalcoholic extract, and the results suggested that it could significantly inhibit xylene-induced ear edema and reduce peritoneal capillary permeability and leukocyte infiltration induced by an intraperitoneal injection of acetic acid (Wang et al., 2015). Similar results were obtained in cells. For example, Sun et al. (2017) found that the *Vaccaria* hypaphorine concentration dependently downregulated the expression of inflammatory cytokine and inflammatory enzyme, and then counteracted the increased phosphorylation of nucleus transfer-related proteins that were induced by inflammation, thereby inhibiting a nuclear factor- (NF-) κB signaling pathway to exert its anti-inflammatory effect in lipopolysaccharide- (LPS-) stimulated Raw 264.7 cells. Osteoclasts are the only cells in the human body that undergo the bone resorption. Inhibiting the differentiation and formation of osteoclasts can effectively inhibit bone loss and osteolysis (Wei et al., 2018). A previous study suggested that *Vaccaria* hypaphorine inhibits the formation, differentiation, and resorption of osteoclast to attenuate inflammatory bone loss in LPS-treated mice by inhibiting an extracellular signal-regulated kinase (ERK), p38, a c-Jun N-terminal kinase (JNK), and an NF-κB p65 phosphorylation (Chen et al., 2018). However, Liu et al. (2019) demonstrated that *Vaccaria* inhibits formation and function of osteoclast *in vivo* and *in vitro*, as well as Ti particle-induced osteolysis. Chronic pelvic inflammatory disease (CPID) refers to chronic inflammation of the female internal organs, surrounding connective tissue, and pelvic peritoneum (Chen, 2012; Bu et al., 2015). Man Pen Fang (MPF), a Chinese herbal compound consisting of the whole plants of *Thlaspi arvense* L. (Cruciferae), *Gleditsia sinensis* Lam. (Fabaceae), *Smilax china* L. (Liliaceae), *Euonymus alatus* (Thunb.) Sieb. (Celastraceae), and the seeds of *V. segetalis* (Neck; Caryophyllaceae), was proven to be effective for treating CPID in the previous studies (Kim et al., 2015; Li et al., 2016). In this formulation, *V. segetalis* (Neck) plays an important role because it has analgesic and anti-inflammatory properties, and activates blood circulation by dissipating blood stasis. In previous studies, Zhang et al. (2017) constructed a CPID mouse model (Tuffrey et al., 1992; Chen et al., 2008) and treated the animals with MPF. They revealed that MPF has a significant dose-dependent anti-inflammatory effect during the CPID treatment, and it also plays a positive role by decreasing the serum levels of inflammatory cytokines such as interleukin- (IL-) 6, IL-10, tumor necrosis factor (TNF)-α, and transforming growth factor (TGF)-β (Zhang et al., 2017). Zhang et al. (2017) conducted mechanistic research on TCMs and provided a theoretical support for the use of MPF to treat CPID. Trichinosis is an infectious disease caused by parasites, and it poses a serious hazard to the pork industry and human health (Rostami et al., 2017). In the host, *Trichinella* activates inflammatory cells to overexpress cyclooxygenase-2 (COX-2; Othman et al., 2016) and other inflammatory factors. Xu et al. (2019) demonstrated that *Vaccaria* n-butanol extract (VNE) from the seeds of *V. segetalis* can significantly relieve the symptoms of *Trichinella spiralis* infection. However, VNE from the seeds of *V. segetalis* did not significantly and directly affect the viability of *T. spiralis* muscle larvae. The study suggested that the survival rate of *T. spiralis* muscle larvae did not differ between the control and VNE treatment. Moreover, VNE exerted anti-inflammatory effects by repressing the IL-1β, IL-6, TNF-α, and COX-2 expression in mice. Therefore, it was speculated that VNE is likely to exert its anti-inflammatory effect by reducing the inflammatory response in infected mice and exhibiting similar insecticidal effects to *T. spiralis* (Xu et al., 2019). Albendazole is commonly used to treat human trichinellosis. However, because of its clinical side effects, VNE may be an adjuvant to the existing drug. A recent study provided insights into the mechanism of the anti-inflammatory effects of *V. segetalis*. Mao found that crude polysaccharides from the seeds of *V. segetalis* (SVCP) can effectively prevent the urinary tract infections that are induced by uropathogenic Escherichia coli (UPEC) in 2020. SVCP-induced upregulation of IL-6 and IL-8 helps to eliminate bacteria in the urine, and does not cause tissue damage and acute pyelonephritis *via* the upregulation of pro-inflammatory cytokines. In addition, the authors found that the application of SVCP could upregulate the PIGR expression in rat kidneys, which was significantly suppressed by UPEC (Mao et al., 2020). This suggests that SVCP can prevent the infection caused by UPEC by stimulating an innate immunity of the kidneys. In addition, (Mao et al., 2021) explored the potential mechanism of SVCP in the treatment of kidney infections in a recent research study. The administration of SVCP upregulated the low expression of Cathelicidin family (LL-37 and CRAMP) in the UPEC-induced kidney infection model, and upregulated the expression of Toll-like receptors (TLRs). TLR agonists can stimulate the expression of cathelicidin (Liu et al., 2006; Vandamme et al., 2012). In summary, SVCP may increase the expression of cathelicidin by activating TLRS to protect the kidney from infection. This enriches the mechanism of action of SVCP.

Currently, antibiotics are the most widely used drugs to treat inflammation-related diseases. However, the long-term use of antibiotics in large quantities will lead to drug resistance and cause serious side effects in some patients, leading to treatment failures. The anti-inflammatory effect of *V. segetalis* provides new avenues for the treatment of inflammatory diseases.

### Anticancer Activity

In 1971, Folkman (1971) first proposed the theory of tumor angiogenesis. Through an in-depth study of malignancies, the researchers found that angiogenesis is an important process in tumorigenesis and tumor development. Angiogenesis can be activated at different stages of tumor development. Currently, anti-angiogenic drugs represent a leading field in the development of new antitumor drugs (Viallard and Larrivee, 2017). The inhibition of angiogenesis has become a necessary strategy for antitumor therapy (D'Amato and Adamis, 1995; Folkman, 1995; Tímár et al., 2001). Continuous proliferation and migration of vascular endothelial cells are the primary steps in angiogenesis. During the neovascularization, endothelial cells first establish a cell-to-cell contact and then proliferate and migrate to a perivascular matrix where cellular connections are re-established and new vessels are formed (Carmeliet and Jain, 2000). Vascular endothelial cells can also secrete vascular growth factors to promote the proliferation of vascular endothelial cells and tumor cells. The inhibition of vascular endothelial cells can inhibit the proliferation of tumor cells and increase apoptosis in metastatic tumor cells. Therefore, anti-angiogenic therapy targeting vascular endothelial cells involved in the proliferation and migration can be an effective antitumor strategy.

Platelet-endothelial cell adhesion molecule-1 (PECAM-1), which is also known as CD31, is a key molecule of cell adhesion. In the process of neovascularization, CD31 is closely related to the movement of endothelial cells, and it may be involved in the signal transduction that is responsible for cell adhesion (Sun et al., 2000). CD31-blocking antibodies inhibit angiogenesis that is induced by cytokines and tumors in different animal models (Lertkiatmongkol et al., 2016).

Many studies have suggested that *V. segetalis* has anticancer and anti-angiogenic effects. We have summarized the pharmacological data and the effects of an herb (Table 3). The results illustrated that *V. segetalis* extract has anticancer effects in various cancer cells. *V. segetalis* extract reduced the expression of CD31 in peripheral endothelial cells and reduced the microvessel density in surrounding tissues. Further research revealed that *V. segetalis* water extract significantly inhibits the proliferation and migration of human mammary epithelial cells (HMECs) in a concentration-dependent manner. This indicates that the main function of *V. segetalis* extract is to block the proliferation and migration of endothelial cells. *V. segetalis* has anticancer effects *in vitro*, and these effects are markedly enhanced in select cancer cells. Other studies demonstrated that *V. segetalis* extract can decrease the formation of abundant microvessels, which was induced by a basic fibroblast growth factor (bFGF). bFGF is found in almost all mesoderm-derived and neuroectoderm-derived tissues as well as the tumors derived from these tissues. bFGF has been shown to have a mitogenic, chemotactic, and angiogenic activity, which promotes cell growth, differentiation, and motility (Finzel et al., 1984). By analyzing a few literature studies, we determined that *V. segetalis* extract can reduce the formation and expansion of tumors by suppressing the formation of abundant microvessels and preventing the proliferation and migration of epithelial cells. Additionally, *V. segetalis* extract will likely to inhibit angiogenesis that is induced by cytokines and tumors by reducing the expression of CD31. Inhibiting angiogenesis was also shown, which often leads to the reduction of tumors; thus, angiogenesis is a critical prerequisite for the tumor formation, and the anti-angiogenic effect of *V. segetalis* provides a new strategy for treating tumors. Anti-angiogenic drugs that are commonly used in clinical practice include vascular endothelial growth factor (VEGF) monoclonal antibodies (Cao et al., 2015), thalidomide (Bladé et al., 2001), and vandetanib (Vitagliano et al., 2010), but these drugs can cause serious side effects and increase medical costs. The discovery and study of anti-angiogenic effects of *V. segetalis* have provided new directions for the development of anticancer drugs in the future.

**Table 3:** Anticancer activity of *V. segetalis*.

	**Model used**	**Plant part used**	**Extract type**	**Admin**	**Dosage/duration**	**Control**	**Results**	**References**
Anti-angiogenesis and anticancer	*In vivo*. Mice, injection of Lewis lung cancer cells	Seeds, *Vaccaria segetalis*	Seeds, decocted twice, filtered, decompressed and condensed into concretes, and freeze-dried to powder	Per oral	Mice were fed with the solution (100 μg/mL) prepared with dry powder after the fourth day of inoculation	Baseline control/Negative control	*Vaccaria segetalis* extract (40 μg) reduced tumors by 58.3%, reduced CD31 expression in peripheral endothelial cells, and reduced the microvessel density in surrounding tissues. It indicated that the extract prevented progress of established tumors and reduced angiogenesis	Feng et al., 2012
	*In vitro*. MTT assay	Seeds, *Vaccaria segetalis*	Seeds, decocted twice, filtered, decompressed and condensed into concretes, and freeze-dried to powder	*In vitro*	Different concentrations (μg/ml)	Negative control	IC_50_ = 50 μg/mL *Vaccaria segetalis* extract inhibited the migration of HMECs in a dose-dependent manner. It suggested that the extract can inhibit the migration of vascular endothelial cells	Feng et al., 2012
	*In vivo*. CAM assay; *In vivo*. Mice, injection of Matrigels.	Seeds, *Vaccaria segetalis*	Seeds, decocted twice, filtered, decompressed and condensed into concretes, and freeze-dried to powder	Injection; Matrigels mixed drugs	Prepare the extract as (100 μg / ml) and treat with 10 μL; 100 μL 100 μg/mL Vaccaria segetalis extract	Negative control; Baseline control/Positive control/negative control	The new blood vessel formation in the extract group was significantly reduced The extract significantly reduced the number of invasive endothelial cells in the Matrigel plug and inhibited microvessel formation	Feng et al., 2012
Anti-angiogenesis and Anticancer	*In vivo*. Mice, subcutaneous injection of H22 cells for solid carcinoma model	Seeds, *Vaccaria segetalis*	Vaccaria segetalis 70% ethanol extract, purified by D-101 resin column.	Intragastric administrate	Treatment group (1, 2.5, and 5 mg/kg)	Negative control/three treatment groups	The results suggested that mouse body weight increased, CD31 expression in tumor vessels decreased, and the apoptosis in tumor cells and vascular endothelial cells was induced. It implied that the use of *Vaccaria segetalis* improved the health of mice, and had the effect of inhibiting tumor growth and anti-angiogenesis	Gao Y. Y. et al., 2010
Anticancer	*In vitro*. Cancer cell line (A549, MCF-7, PC-3, LNCaP) Normal human mammary epithelial cells (HMECs); Antiproliferative assay	Seeds, *Vaccaria segetalis*	*Vaccaria segetalis* water extract	*In vitro*.	Different concentration (μg/ml)	Treatment groups	The result suggested that *Vaccaria segetalis* had anticancer effects *in vitro* and these effects are markedly greater in various cancer cells This experiment indicated that *Vaccaria segetalis* water extract can inhibit proliferation of cancer cell lines	Mark et al., 2005
Anti-angiogenesis	*In vivo*. Mice, injection of Matrigels.	Seeds, *Vaccaria segetalis*	Seeds, decocted twice, filtered, decompressed and condensed into concretes, and freeze-dried to powder	Matrigels mixed drugs	0.1 g extract	Negative control/Positive control	Reduction of endothelial cells and decrease of the formation of abundant microvessels induced by basic fibroblast growth factor (bFGF)	Passaniti et al., 1992
Anticancer by promoting apoptosis of cancer cells	*In vitro*. MTT assay/DNA fragmentation; *In vivo*. Mice, HepG2 xenograft Kunming	Seeds, *Vaccaria segetalis*	Segetoside I, standardized by crude ethanol extract, CH2Cl2/MeOH mixture, MeOH–H2O (RP-8 and RP-18 columns) gradient elution	*In vitro*; Intraperitoneal injection	Different concentration of segetoside I; Segetoside I (1.25, 2.5, 5 mg/kg)	Negative control/Positive control	IC_50_= 8.62 μM; Dose-dependent DNA fragmentation and increase Bax/Bcl-2 expressions by Segetoside I (0.82, 8.2, 82 μM); Dose-dependent inhibition of tumor growth with segetoside I	Firempong et al., 2016

Tumors are caused by the acceleration of cell proliferation and suppression of apoptosis. Tumor treatment can also be accomplished by inducing apoptosis in tumor cells. Many studies support the apoptotic effect of *V. segetalis* in cancer cells. The results identified the *V. segetalis* compounds that have significant antitumor effects, and these are triterpenoid saponins (segetoside H and segetoside I) and some unknown compounds (Gao Y. Y. et al., 2010; Balsevich et al., 2012). Segetosides H and I, which are isolated from *V. segetalis*, have significant anticancer effects (Table 3; Yun et al., 1998). Previous studies demonstrated that segetoside I can induce DNA fragmentation and the upregulation of apoptosis-related genes, suggesting that the activation of apoptotic signaling events may have been initiated. Segetoside I also suppressed hepatic tumor growth in mice with virtually no cytotoxicity, and prolonged animal survival (Firempong et al., 2016), and thus, it may be an effective candidate for treating tumors. These results suggest that segetosides H and I can induce cancer cell apoptosis by activating apoptotic pathways to prevent the development of cancer, which may make them valuable antitumor chemotherapeutics.

In summary, we propose that *V. segetalis* extract can exert its anticancer effects in two ways. First, the extract can inhibit the tumor growth and angiogenesis around the tumor and second, it can activate apoptosis pathways in cancer cells. To study the mechanism of the anticancer action of *V. segetalis* in the future, we should focus on angiogenesis-related and apoptosis-related pathways. Although *V. segetalis* has been used as traditional Chinese herbal medicine for more than 2000 years, its anticancer effect has been newly proposed in recent years and the current experiments remain in the preclinical stage, including cell and animal models. Deeper mechanism studies and more expansive clinical data are needed to prove and support medication methods, dosages, and other variables. Thus, much work remains before *V. segetalis* or its extracts can be used to treat patients with cancer.

### Inhibition of Apoptosis

Apoptosis is also known as a programmed cell death. Despite its ability to induce apoptosis in tumor cells as mentioned previously, *V. segetalis* can inhibit apoptosis that is caused by apoptotic factors in normal cells. Vaccarin was shown to exert a potential protective effect in H_2_O_2_-injured human EA.hy926 endothelial cells by inhibiting the Notch signaling pathway and downregulating caspase-3, which has a dominant role in the execution of the apoptotic process. Caspase-3 activation is a central link in apoptosis (Xie et al., 2015). H_2_O_2_ is often used to develop a validation model, and it stimulates cells to produce reactive oxygen species (ROS) (Schieber and Chandel, 2014). Previous studies revealed that the ROS production and apoptosis can occur simultaneously (Corbacho et al., 2002). Further studies illustrated that vaccarin can suppress a high glucose-induced damage in EA.hy926 cells, which was shown by improved cell viability and migratory ability, and this treatment effectively suppressed the caspase-3 overexpression (Qiu et al., 2016).

The abovementioned studies demonstrated that vaccarin, which is an active ingredient of *V. segetalis*, can downregulate the apoptotic gene expression that is induced by apoptotic factors, thereby inhibiting apoptosis. We can imagine a clinical role for vaccarin as an antioxidant based on its ability to prevent H_2_O_2_-induced apoptosis, specifically as a daily supplement.

### Dilation of Blood Vessels

Vasodilators can be used to treat hypertension, coronary atherosclerotic heart disease, angina pectoris, and cerebrovascular sclerosis. *V. segetalis* is often used to treat headache, hypertension, asthmatic pneumonia, and other diseases. Because of its vasodilatory effects, *V. segetalis* (Neck) Garcke decoction had a significant concentration-dependent relaxing effect on the norepinephrine-induced precontraction of the rabbit aortic smooth muscle. After removing endothelial cells, the relaxation effect of *V. segetalis* (Neck) Garcke on noradrenaline-precontracted arterial rings was significantly decreased (Hua'e et al., 2007). Morita et al. (1997a) found that segetalins A, D, F, G, and H exhibited vasodilatory activity against norepinephrine-induced aortic contraction in rats, and among these, segetalins G and H had the strongest diastolic activity. In addition, a study by Jing et al. (2007) reported the same relaxation in aortic ring samples that lacked an endothelium. The volatile oil in *V. segetalis* may be responsible for this function (Shiva Kumar et al., 2017). The specific mechanisms of segetalin action on vasodilation and contractile activity require further investigation. Previously, we explained that TCM often uses the seeds of *V. segetalis* to mechanically stimulate auricular points to assist in the treatment of hypertension. In addition to the unique characteristics of its seeds, we speculate that it is also possible that the volatile oil of *V. segetalis* could enter the body through the skin at the ear point. (Morita et al., 2006) found that the segetalins in *V. segetalis* extract have a vasodilatory activity. Although the vasodilatory activity of segetalins has been reported in animal models, there are no clinical data to date. However, the evidence points to a new strategy for the treatment of vasoconstriction-related diseases.

### Promotion of Lactation

A recent study indicated that the use of *V. segetalis* during breastfeeding to promote breast milk is common in Macau (Zheng et al., 2020). Many studies have revealed that *V. segetalis, Leonurus*, and *Astragalus* can significantly promote lactation in female rats (Baoming and Anshan, 2007). Although this treatment remains in use, the mechanism by which *V. segetalis* induces lactation is unclear.

One of the primary ingredients in *V. segetalis* is vaccarin (Jia et al., 1998), which promotes the proliferation of mammary epithelial cells and enhances their secretory function (Leonoudakis et al., 2010). Mammary epithelial cells are the breast biological generators that synthesize and secrete milk. The number and activity of mammary epithelial cells are closely related to the lactation performance of livestock (Planas-Silva et al., 2006). The latest research has enriched the lactation mechanism of Vaccarin. Vaccarin and PRL have similar effects in stimulating the proliferation of breast epithelial cells and enhancing their secretory function, the expression of Cylin D1, the phosphorylation of mechanistic target of rapamycin (mTOR), and the regulation of the expression of a sterol regulatory element binding protein 1c (SREBP-1c). In addition, Vaccarin can promote the expression of PRL receptors. In summary, Vaccarin can promote the breast epithelial cell proliferation and enhance its secretion ability *via* the PRL receptor-PI3K-Cyclin D1/SREBP-1c/mTOR signaling pathways (Yu et al., 2020). JAK2 is important for the PRL signal transduction and normal breast tissue development, and STAT5a is necessary for the breast development (Gass et al., 2003). β-casein is an important milk protein, and its secretion level reflects the lactation ability of breast epithelial cells to a certain extent. Gao X. J. et al. (2010) found that the dibutyl phthalate (DBP) injection can increase the milk production in cows. Liu et al. (2010) found that DBP could significantly increase the proliferation and viability of mammary epithelial cells in dairy cows. It was also found that DBP could increase the β-casein expression and lactose secretion in breast epithelial cells (Tong et al., 2013). In addition, Tong et al. found that DBP from *V. segetalis* can promote lactation and the proliferation of breast epithelial cells and activate a JAK-STAT5-signaling pathway by increasing the STAT5 phosphorylation levels (Tong et al., 2011). These studies demonstrated that DBP has estrogenic activity, and that it can activate a lactation-signaling pathway and promote lactation by regulating the milk protein, lactose, and milk fat synthesis. However, as a highly toxic substance, the dosage and application of DBP require more theoretical research and experimental support.

The low postpartum lactation ability of mammals is mainly attributable to the insufficient activation of the relevant endocrine system in the body, preventing mammary glands from producing milk normally and hindering milk excretion. Chinese herbal medicine can promote lactation by regulating the metabolism of postpartum mammals and increasing endocrine levels *in vivo*. Kleinberg and Ruan (2008) found that *V. segetalis* increased the content of growth hormones (GHs) and PRL in serum from dairy cows, which in turn promoted the breast development and improved the lactation performance. Jun-Xian et al. (2013) used *V. segetalis* water extract, which had a similar effect to estrogen and PRL, and its components could bind to an estrogen receptor to activate a STAT5-signaling pathway, thereby promoting the P-STAT5 expression and milk protein synthesis. Bryant (2009) found that the PRL promoter transcription and PRL synthesis can be induced by stimulating the estrogen receptor system and pituitary transcription factor Pit-1. PRL controls the regulation of milk protein at the translational level, thereby promoting the synthesis of milk proteins. Another study reported similar results and suggested that *V. segetalis* has a specific estrogen-like effect; specifically, its components can bind to an estrogen receptor, and thereby promoting the synthesis of PRL (Itokawa et al., 1995).

Based on these findings, we concluded that the stimulatory effects of *V. segetalis* on the production of milk occur through DBP and that DBP has PRL-like effects. In addition, a few studies have found that the other substances in *V. segetalis* have estrogen-like effects, and estrogen-like active ingredients can bind with estrogen receptors to promote the synthesis of PRL. This implies a synergy between the actions of various substances in *V. segetalis*. These results provide new ideas for future studies on *V. segetalis*.

### Estrogen-Like Action

Many studies have revealed the estrogen-like effect *of V. segetalis*. Pakoussi et al. (2018) found that phytoestrogens enhance uterine contractions. Morita et al. (1997c) discovered vaccaroid A in *V. segetalis* and found that it has a role in promoting uterine contractions. Moreover, the research has illustrated that segetalins A, B, G, and H are cyclic hexapeptides and pentapeptides with estrogenic activity in ovariectomized rats (Morita et al., 1993, 1995; Yun et al., 1997). Segetalins are the only natural cyclic peptides that were reported to have estrogenic activity. When they are digested into acyclic segetalins by enzymes, they lose their activity, indicating that their conformation plays an important role in their function (Morita et al., 1997a). A study found that segetalins G and H at a dose of 2.5 mg/kg significantly increased uterine weight vs. the control (*p* < 0.01) in ovariectomized rats that did not receive the estrogen supplementation for 2 weeks (Yun et al., 1997). Itokawa et al. (1995) applied segetalins A and B to ovariectomized rats for 14 consecutive days and observed similar effects to those of segetalins G and H, verifying that segetalins A, B, G, and H have estrogen-like effects. Ovariectomy-induced bone loss in rats and postmenopausal bone loss in women share many similar features, and the clinical symptoms are often treated by 17β-estradiol supplementation (Kalu, 1991). To study the estrogen-like activity of *V. segetalis* and facilitate its future clinical applications, Shih et al. (2009) constructed an ovariectomized rat model to simulate postmenopausal bone loss in women. In the ovariectomized group, the calcium content of the femur and the fourth lumbar vertebra was significantly reduced, whereas these effects were alleviated by the supplementation with 17β-estradiol or *V. segetalis* extract. *V. segetalis* extract does not cause side effects such as uterine or vaginal hypertrophy. This provides a new direction for the treatment of osteoporosis. Moreover, because of its estrogen-like activity, *V. segetalis* can be used as an alternative to estrogen for people with allergies to synthetic estrogens. In addition, *V. segetalis* can avoid the side effects of estrogen supplementation.

## Toxicology Research

Several toxicological studies related to *V. segetalis* have been conducted. In one study, *V. segetalis* extract obtained *via* a reflux with 75% ethanol was used to treat mice. The minimum toxic amount of *V. segetalis* extract in mice was 100 mg/kg, and the minimum lethal dose was 1,500 mg/kg. *V. segetalis* extract was more toxic to the heart and kidneys of mice at 1,000 mg/kg (approximately lethal dose) than lung, and there was no serious functional damage in mice treated with 200 mg/kg of *V. segetalis* extract. Therefore, *V. segetalis* shows a good safety profile, and it could be a promising therapeutic modality (Gao et al., 2013).

Segetoside I is an extract of *V. segetalis*, and it has been a focus of research. One study showed that intraperitoneal segetoside I in mice had an LD50 (median lethal dose) of 14.5 mg/kg (Firempong et al., 2016). The mice were treated with segetoside I at different concentrations and sacrificed after 14 days, and their respective organ coefficients were determined. The tissue coefficients of the heart, liver, and kidney did not change significantly, whereas the tissue coefficient of the spleen increased with increasing doses of segetoside I. The spleen coefficient was significantly improved by segetoside I at the concentration of 1.25, 2.5, and 5 mg/kg. This demonstrated that segetoside I did not significantly affect the body weight of mice, but that it damaged the spleen at doses exceeding 5 mg/kg. In addition, the studies have explored the cytotoxic effects of different extracts of *V. segetalis* in different cells, and IC50 was obtained (Table 4; Ma et al., 2008).

**Table 4:** Cytotoxic activity (IC50, mM) of compounds 1–6.

**Cell lines**	**1**	**2**	**3**	**4**	**5**	**6**	***Topotecan***	***Docetaxel***
LNcap	3.6	3.4	2.5	4.2	12.9	1.2	0.053	
A-549	1.0	3.0	11.0	1.0	7.2	0.4		<0.01
P-388	0.8	9.4	0.7	3.7	1.6	0.1		<0.01

*Compound 1, vaccaroside I; Compound 2, vaccaroside E; Compound 3, vaccaroside G; Compound 4, vaccaroside B; Compound 5, segetoside H; Compound 6, segetoside I*.

*LNCaP, human prostate cancer cell line; A549, human lung cancer cell line; P388, mouse leukemia cell line*.

*Topotecan and docetaxel were used as positive controls*.

In a previous study, it was mentioned that *V. segetalis* has an anti-inflammatory effect, but at the same time it has an estrogen-like effect, which also leads to certain side effects. Overdose of estrogen by men who have not entered puberty will cause the secondary sex characteristics of men to not appear but the second sex characteristics of women; excessive estrogen in puberty men will cause a gradual decline of the second sex characteristics of men, in addition to causing endocrine disorders, obesity, and even affect fertility (Kabir et al., 2015). This has led to a clinical application of Wang Buliuxing more oriented to females, and whether Wang Buliuxing can be used for the treatment of males and the applied dosage need more literature support. In addition, the anti-inflammatory effect of Wang Buliu Xing may hide an inflammatory process caused by the microorganism infection, which may lead to an aggravation of the infection, and thus may not fundamentally solve the problem.

Taken together, the toxicological information on *V. segetalis* remains extremely limited, and further research is required to determine the extraction, purification, and application of the active ingredients of *V. segetalis*.

## Conclusion

*V. segetalis* is a plant used in traditional Chinese herbal medicine that occupies an extremely important position in the Chinese medicine theory. With an increasing amount of research on the active ingredients of *V. segetalis, V. segetalis* could be used with modern medicine to treat blood strangury, lactation deficiencies, and chylorrhea. *V. segetalis* can also be used to treat hypertension, headache, and gallstones through its unique ability to physically stimulate the corresponding acupuncture points on the ear or body. This article has provided in detail the chemical and pharmacological properties of *V. segetalis* and summarized the recent literature studies. The main ingredients in *V. segetalis* include flavonoids, cyclic peptides, saponins, and polysaccharides, but several active substances have not been identified, indicating the need for a more systematic phytochemical research. In terms of its pharmacological effects, *V. segetalis* has anti-inflammatory, anti-angiogenic, anticancer, anti-apoptosis, and estrogenic effects, dilates blood vessels, and promotes lactation. Many diseases are treated poorly using modern medicine, and traditional and complementary medicine can provide new treatment modalities for these diseases.

Although substantial research has been conducted on *V. segetalis*, many unknown elements in *V. segetalis* remain. In addition, some studies used hydroalcoholic crude extracts instead of the pure active ingredients of *V. segetalis* as the experimental objects. We speculate that hydroalcoholic crude extracts are used because the extraction process for the active ingredients of *V. segetalis* is more complicated. In addition, we found that *V. segetalis* crude extracts and some effective active ingredients also have anticancer effects that have not been mentioned in clinical applications, thereby further enhancing the significance of *V. segetalis* in modern research and providing new directions of future research.

Future research on *V. segetalis* should specifically focus on the four issues that are described below. First, we should continue to explore the unknown elements in *V. segetalis*. Second, the processes for extracting the effective active substances of *V. segetalis* should be further improved. Third, the mechanisms of the anti-inflammatory, anticancer, and anti-apoptotic effects of *V. segetalis* should be further elucidated. Finally, more research on the pharmacology and toxicology is needed to provide additional evidence for clinical applications.

## Author Contributions

CL, XZ, and MT conceptualized the study. MT, YH, XW, TC, CY, NW, ZZ, and BZ investigated the study. MT wrote original draft preparation. CL and XZ helped in writing and editing the review. All authors contributed to the article and approved the submitted version.

## Conflict of Interest

The authors declare that the research was conducted in the absence of any commercial or financial relationships that could be construed as a potential conflict of interest.

## Abbreviations

PRL: Prolactin
LPS: Lipopolysaccharide
ERK: Extracellular signal-regulated kinases
JNK: C-Jun N-terminal kinase
CPID: Chronic pelvic inflammatory disease
MPF: Man-Pen-Fang
IL-6: Interleukin-6
TNF-α: Tumor necrosis factor-α
TGF-β: Transforming growth factor-β
COX-2; VNE: Cyclooxygenase-2; Vaccaria n-butanol extract
PECAM-1: Platelet-endothelial cell adhesion molecule-1
A549: Human; lung carcinoma
Panc-1: Human pancreatic carcinoma
MCF-7: Human breast adenocarcinoma
PC-3: Human prostate adenocarcinoma
LNCaP: Human prostate carcinoma
HMECs: Human mammary epithelial cells
CAM: The chick chorioallantoic membrane
bFGF: basic fibroblast growth factor
VEGF: Vascular endothelial growth factor
HepG2: Human hepatoma cells
ROS: Reactive oxygen species
DBP: Dibutyl phthalate
GH: Growth hormone
IGF: Insulin-like growth factor.

## References

[B1] Aswad M. Rayan M. Abu-Lafi S. Falah M. Raiyn J. Abdallah Z.. (2018). Nature is the best source of anti-inflammatory drugs: indexing natural products for their anti-inflammatory bioactivity. Inflamm. Res. 67, 67–75. 10.1007/s00011-017-1096-5 28956064

[B2] Balsevich J. J. Ramirez-Erosa I. Hickie R. A. Dunlop D. M. Bishop G. G. Deibert L. K. (2012). Antiproliferative activity of *Saponaria vaccaria* constituents and related compounds. Fitoterapia 83, 170–181. 10.1016/j.fitote.2011.10.010 22056663

[B3] Banno N. Akihisa T. Tokuda H. Yasukawa K. Taguchi Y. Akazawa H.. (2005). Anti-inflammatory and antitumor-promoting effects of the triterpene acids from the leaves of *Eriobotrya japonica*. Biol. Pharm. Bull. 28, 1995–1999. 10.1248/bpb.28.1995 16204964

[B4] Baoming S. Anshan S. (2007). Effects of Chinese herbs on lactation of rat and performance of offspring. J. North. Agric. Univer. 14, 22–26. CNKISUNDBYN.0.2007-01-006

[B5] Bernard P. Scior T. Didier B. Hibert M. Berthon J. Y. (2001). Ethnopharmacology and bioinformatic combination for leads discovery: application to phospholipase A(2) inhibitors. Phytochemistry 58, 865–874. 10.1016/S0031-9422(01)00312-0 11684183

[B6] Bladé J. Esteve J. Rosi?ol L. Perales M. Montoto S. Tuset M.. (2001). Thalidomide in refractory and relapsing multiple myeloma. Semin. Oncol. 28, 588–592. 10.1016/S0093-7754(01)90029-6 11740814

[B7] Bozak K. R. Yu H. Sirevg R. Christoffersen R. E. (1990). Sequence analysis of ripening-related cytochrome P-450 cDNAs from avocado fruit. Proc. Natl. Acad. Sci. U.S.A. 87, 3904–3908. 10.1073/pnas.87.10.3904 1692626 PMC54012

[B8] Bryant W. (2009). Environmental estrogens stimulate gene transcription in the prolactin promoter. Int. J. Biol. 2, 35–43. 10.5539/ijb.v2n1p35

[B9] Bu X. Liu Y. Lu Q. Jin Z. (2015). Effects of “danzhi decoction” on chronic pelvic pain, hemodynamics, and proinflammatory factors in the murine model of sequelae of pelvic inflammatory disease. Evid. Based Complement. Alternat. Med. 2015:547251. 10.1155/2015/547251 27087818 PMC4806651

[B10] Cao R. Zhang S. Ma D. Hu L. (2015). A multi-center randomized phase II clinical study of bevacizumab plus irinotecan, 5-fluorouracil, and leucovorin (FOLFIRI) compared with FOLFIRI alone as second-line treatment for Chinese patients with metastatic colorectal cancer. Med. Oncol. 32:325. 10.1007/s12032-014-0325-9 25481673

[B11] Carmeliet P. Jain R. K. (2000). Angiogenesis in cancer and other diseases. Nature 407, 249–257. 10.1038/35025220 11001068

[B12] Chen H. Guo T. Wang D. Qin R. (2018). Vaccaria hypaphorine impairs RANKL-induced osteoclastogenesis by inhibition of ERK, p38, JNK and NF-κB pathway and prevents inflammatory bone loss in mice. Biomed. Pharmacother. 97, 1155–1163. 10.1016/j.biopha.2017.11.044 29136954

[B13] Chen K. J. (2012). Blood stasis syndrome and its treatment with activating blood circulation to remove blood stasis therapy. Chin. J. Integrat. Med. 18, 891–896. 10.1007/s11655-012-1291-5 23238996

[B14] Chen Y. Liu Q. Chen Y. Huang W. Jin B. Ding Z.. (2017). Study on hypoglycemic effects of total flavonoid aglycon from leaves of Carya cathayensis. Chin. Arch. Tradit. Chin. Med. 35, 2033–2035. CNKISUNZYHS.0.2017-08-028

[B15] Chen Y. Tian L. Wang X. Wang C. X. (2008). Drug particles of FUYANNING to chronic pelvic inflammatory disease model of the impact of rats' IL-2 IL-6. Chin. Arch. Tradit. Chin. Med.

[B16] Choi D. W. Jung J. D. Ha Y. I. Park H. W. Dong S. I. Chung H. J.. (2005). Analysis of transcripts in methyl jasmonate-treated ginseng hairy roots to identify genes involved in the biosynthesis of ginsenosides and other secondary metabolites. Plant Cell Rep. 23, 557–566. 10.1007/s00299-004-0845-4 15538577

[B17] Corbacho A. M. Martinez De La Escalera G. Clapp C. (2002). Roles of prolactin and related members of the prolactin/growth hormone/placental lactogen family in angiogenesis. J. Endocrinol. 173, 219–238. 10.1677/joe.0.1730219 12010630

[B18] Dahiya R. Dahiya S. (2021). Natural cyclic polypeptides as vital phytochemical constituents from seeds of selected medicinal plants. Arch. Pharm. Chem. Life Sci. 354:e2000446. 10.1002/ardp.202000446 33522644

[B19] Dahiya R. Kaur K. (2008). Synthesis and pharmacological investigation of segetalin C as a novel antifungal and cytotoxic agent. Arzneimittelforschung 58, 29–34. 10.1055/s-0031-1296463 18368948

[B20] D'Amato R. J. Adamis A. P. (1995). Angiogenesis inhibition in age-related macular degeneration. Ophthalmology 102, 1261–1262. 10.1016/S0161-6420(95)30876-7 9097761

[B21] Feng L. Zhang X. Hua H. Qiu L. Zhang L. Lv Z. (2012). *Vaccaria segetalis* extract can inhibit angiogenesis. Asian Biomed. 6, 683–692. 10.5372/1905-7415.0605.108

[B22] Finzel B. C. Poulos T. L. Kraut J. (1984). Crystal structure of yeast cytochrome c peroxidase refined at 1.7-A resolution. J. Biol. Chem. 259, 13027–13036. 10.1016/S0021-9258(18)90651-4 6092361

[B23] Firempong C. K. Zhang H. Y. Wang Y. Chen J. Cao X. Deng W.. (2016). Segetoside I, a plant-derived bisdesmosidic saponin, induces apoptosis in human hepatoma cells *in vitro* and inhibits tumor growth *in vivo*. Pharmacol. Res. 110, 101–110. 10.1016/j.phrs.2016.04.032 27180010

[B24] Folkman J. (1971). Tumor angiogenesis: therapeutic implications. N. Engl. J. Med. 285, 1182–1186. 10.1056/NEJM197111182852108 4938153

[B25] Folkman J. (1995). Angiogenesis in cancer, vascular, rheumatoid and other disease. Nat. Med. 1, 27–31. 10.1038/nm0195-27 7584949

[B26] Gao X. J. Tong H. L. Li-Min L. U. Qing-Zhang L. I. (2010). Preparation of dibutyl phthalate isomer from Semen Vaccariae and its influence on milk production and milk quality of dairy cow. China Dairy Indust. 2010, 36–37. 10.3969/j.issn.1001-2230.2010.04.011

[B27] Gao Y. Y. Feng L. Qiu L. Y. (2013). Research on acute toxicology of *Vaccaria segetalis* extract. Guangzhou Chem. Indust. 41, 17–21. 10.3969/j.issn.1001-9677.2013.19.003

[B28] Gao Y. Y. Qiu L. Y. Kang X. X. Wang H. Jin J. (2010). “AntiTumor effect and its mechanism of vaccaria segetalis on mouse inoculated H22 solid carcinoma,” in International Conference on Bioinformatics & Biomedical Engineering WuXi. 10.1109/ICBBE.2010.5517710

[B29] Gass S. Harris J. Ormandy C. Brisken C. (2003). Using gene expression arrays to elucidate transcriptional profiles underlying prolactin function. J. Mammary Gland Biol. Neoplasia 8, 269–285. 10.1023/B:JOMG.0000010029.85796.63 14973373

[B30] Guan F. Q. Liu M. Yu S. Chen Y. Zhao Y. Y. Wang M.. (2013). The antioxidant activity evaluations of the triterpene saponins from *Lonicera macranthoides in vitro*. Lishizh. Med. Mater. Med. Res. 24, 1315–1317. 10.3969/j.issn.1008-0805.2013.06.013

[B31] Güçlü-Ustündag O. Mazza G. (2007). Saponins: properties, applications and processing. Crit. Rev. Food Sci. Nutr. 47, 231–258. 10.1080/10408390600698197 17453922

[B32] Haralampidis K. Trojanowska M. Osbourn A. E. (2002). Biosynthesis of triterpenoid saponins in plants. Adv. Biochem. Eng. Biotechnol. 75, 31–49. 10.1007/3-540-44604-4_2 11783842

[B33] Hua'e J. Caiqin N. Jianmin H. Tuanxiao Z. (2007). Vasodilatation effects of the water decoction of vaccaria segetalis(neck)garcke on rabbit aorta *in vitro*. J. Sichu. Tradit. Chin. Med. 25, 13–15.

[B34] Hui Y. Jun-Li H. Chuang W. (2019). Anti-oxidation and anti-aging activity of polysaccharide from *Malus micromalus* makino fruit wine. Int. J. Biol. Macromol. 121, 1203–1212. 10.1016/j.ijbiomac.2018.10.096 30342941

[B35] Itokawa H. Yun Y. Morita H. Takeya K. Yamada K. (1995). Estrogen-like activity of cyclic peptides from *Vaccaria segetalis* extracts. Planta Med. 61, 561–562. 10.1055/s-2006-959373 8824953

[B36] Jia Z. Koike K. Kudo M. Li H. Nikaido T. (1998). *Triterpenoid saponins* and sapogenins from *Vaccaria segetalis*. Phytochemistry 48, 529–536. 10.1016/S0031-9422(97)01128-X 9654779

[B37] Jing H. Of D. Medicine W. First T. (2007). Vasodilatation effects of the water decoction of *Vaccaria segetalis*(neck) garcke on rabbit aorta *in vitro*. J. Sichu. Tradit. Chin. Med. 25, 13–15. 10.3969/j.issn.1000-3649.2007.08.008

[B38] Jin-Ling H. U. Hong H. U. Yang L. (2014). Studies on the chemical constituents from the seeds of *Vaccaria segetalis*. J. Pharmaceut. Res. 33, 71–72.12512436

[B39] Jun-Xian L. U. Gao Y. S. Tang M. J. Jun-Hua P. U. Zhang X. Y. Qing-Lian G. E. (2013). Effects of free gossypol on immunity of the HISEX young hens. China Anim. Husb. Vet. Med. 40, 118–121. 10.3969/j.issn.1671-7236.2013.04.026

[B40] Kabir E. R. Rahman M. S. Rahman I. (2015). A review on endocrine disruptors and their possible impacts on human health. Environ. Toxicol. Pharmacol. 40, 241–258. 10.1016/j.etap.2015.06.009 26164742

[B41] Kalu D. N. (1991). The ovariectomized rat model of postmenopausal bone loss. Bone Miner. 15, 175–191. 10.1016/0169-6009(91)90124-I 1773131

[B42] Kim Y. Koh J. H. Ahn Y. J. Oh S. Kim S. H. (2015). The Synergic anti-inflammatory impact of *Gleditsia sinensis* Lam. and *Lactobacillus brevis* KY21 on intestinal epithelial cells in a DSS-induced colitis model. Korean J. Food Sci. Anim. Resourc. 35, 604-610. 10.5851/kosfa.2015.35.5.604 26761887 PMC4670888

[B43] Kleinberg D. L. Ruan W. (2008). IGF-I, GH, and sex steroid effects in normal mammary gland development. J. Mammary Gland Biol. Neoplas. 13, 353–360. 10.1007/s10911-008-9103-7 19034633

[B44] Koike K. Jia Z. Nikaido T. (1998). Triterpenoid saponins from *Vaccaria segetalis*. Phytochemistry 47, 1343–1349. 10.1016/S0031-9422(97)00707-3 9611829

[B45] Kozlowska A. Szostak-Wegierek D. (2014). Flavonoids–food sources and health benefits. Rocz. Panstw. Zakl. Hig. 65, 79–85. 25272572

[B46] Leonoudakis D. Singh M. Mohajer R. Mohajer P. Fata J. E. Campbell K. P.. (2010). Dystroglycan controls signaling of multiple hormones through modulation of STAT5 activity. J. Cell Sci. 123, 3683–3692. 10.1242/jcs.070680 20940259 PMC2964112

[B47] Lertkiatmongkol P. Liao D. Mei H. Hu Y. Newman P. J. (2016). Endothelial functions of platelet/endothelial cell adhesion molecule-1 (CD31). Curr. Opin. Hematol. 23, 253–259. 10.1097/MOH.0000000000000239 27055047 PMC4986701

[B48] Li F. He T. Xu Q. Lin L. T. Li H. Liu Y.. (2015). What is the acupoint? A preliminary review of acupoints. Pain Med. 16, 1905–1915. 10.1111/pme.12761 25975413

[B49] Li K. K. Zhou X. Wong H. L. Ng C. F. Fu W. M. Leung P. C.. (2016). *In vivo* and *in vitro* anti-inflammatory effects of Zao-Jiao-Ci (the spine of Gleditsia sinensis Lam.) aqueous extract and its mechanisms of action. J. Ethnopharmacol. 192, 192–200. 10.1016/j.jep.2016.07.020 27401288

[B50] Liang X. Xiu-jun J. (2013). Observation on the therapeutic effect of auricular point pressing bean on 30 cases of allergic rhinitis. J. N. Chin. Med. 45, 181–182. CNKISUNREND.0.2013-11-092

[B51] Li-Fan Liang J. Y. (2007). Research progress of *Vaccaria segetalis*. Strait Pharm. J. 19, 1–1.

[B52] Liu J. Limin L. Xiaofei L. Qingzhang L. (2010). Effects on the proliferation and lactation ability of dairy cow mammary gland epithelial cells by semen vaccariae active isomer. Key Lab. Dairy Sci. Educ. 33, 66–68.

[B53] Liu P. T. Stenger S. Li H. Wenzel L. Tan B. H. Krutzik S. R.. (2006). Toll-like receptor triggering of a vitamin D-mediated human antimicrobial response. Science 311, 1770–1773. 10.1126/science.1123933 16497887

[B54] Liu Y. (2018). Auricular pressing therapy in the treatment of hypertension for 30 cases. Chin. Med. Mod. Dist. Educ. China. 16:127–128

[B55] Liu Y. Song F. M. Ma S. T. Moro A. Feng W. Y. (2019). Vaccarin prevents titanium particle-induced osteolysis and inhibits RANKL-induced osteoclastogenesis by blocking NF-kappaB and MAPK signaling pathways. J. Cell Physiol. 234, 13832–13842. 10.1002/jcp.28063 30637734 PMC13483030

[B56] Ma C. H. Fan M. S. Lin L. P. Tang W. D. Lou L. G. Ding J.. (2008). Cytotoxic triterpenoid saponins from *Vaccaria segetalis*. J. Asian Nat. Prod. Res. 10, 177–184. 10.1080/10286020701394381 18253886

[B57] Mao G. H. Ren Y. Li Q. Wu H. Y. Jin D. Zhao T.. (2016). Anti-tumor and immunomodulatory activity of selenium (Se)-polysaccharide from Se-enriched *Grifola frondosa*. Int. J. Biol. Macromol. 82, 607–613. 10.1016/j.ijbiomac.2015.10.083 26522247

[B58] Mao X. Guo H. Yao R. Bao L. Sun J. Bao Y.. (2020). Crude polysaccharides from the seeds of Vaccaria segetalis prevent the urinary tract infection through the stimulation of kidney innate immunity. J. Ethnopharmacol. 260:112578. 10.1016/j.jep.2020.112578 31962152

[B59] Mao X. Yao R. Guo H. Bao L. Bao Y. Xu Y.. (2021). Polysaccharides extract from Vaccaria segetalis seeds inhibits kidney infection by regulating cathelicidin expression. J. Ethnopharmacol. 267:113505. 10.1016/j.jep.2020.113505 33141055

[B60] Mark Shoemaker M. Hamilton B. Dairkee S. H. Cohen I. Campbell M. J. (2005). In vitro anticancer activity of twelve Chinese medicinal herbs. Phytother. Res. 19:649–51. 10.1002/ptr.1702 16161030

[B61] Minliangzou Z. Ainalao S. (1999). Segetoside L, a new triterpenoid saponin from *Vaccaria segetalis*. ??????(???) 15, 55–57.

[B62] Min-ying T. (2005). 59 cases of herpes zoster treated with Vaccaria segetalis. Chin. J. Rural Med. Pharm. 13, 51–51. CNKISUNXCYY.0.2006-06-053

[B63] Morita H. Eda M. Iizuka T. Hirasawa Y. Sekiguchi M. Yun Y. S.. (2006). Structure of a new cyclic nonapeptide, segetalin F, and vasorelaxant activity of segetalins from *Vaccaria segetalis*. Bioorgan. Med. Chem. Lett. 16, 4458–4461. 10.1016/j.bmcl.2006.06.083 16844371

[B64] Morita H. Nagashima S. Takeya K. Itokawa H. (1993). Astins A and B, antitumor cyclic pentapeptides from *Aster tataricus*. Chem. Pharm. Bull. 41, 992–993. 10.1248/cpb.41.992 8339347

[B65] Morita H. Yun Y. S. Takeya K. Itokawa H. (1997a). Conformational preference for segetalins G and H, cyclic peptides with estrogen-like activity from seeds of Vaccaria segetalis. Bioorgan. Med. Chem. 5, 2063–2067. 10.1016/S0968-0896(97)00135-1 9416423

[B66] Morita H. Yun Y. S. Takeya K. Itokawa H. Shiro M. (1995). Conformational analysis of a cyclic hexapeptide, segetalin A from *Vaccaria segetalis*.? Tetrahedron 51, 5987–6002. 10.1016/0040-4020(95)00277-F

[B67] Morita H. Yun Y. S. Takeya K. Itokawa H. Shirota O. (1997b). Thionation of segetalins A and B, cyclic peptides with estrogen-like activity from seeds of Vaccaria segetalis. Bioorgan. Med. Chem. 5, 631–636. 10.1016/S0968-0896(97)00001-1 9113340

[B68] Morita H. Yun Y. S. Takeya K. Itokawa H. Yamada K. Shirota O.. (1997c). A new triterpenoid saponin with contractility of rat uterine from Vaccaria segetalis. Bioorg. Med. Chem. Lett. 7, 1095–1096. 10.1016/S0960-894X(97)00168-6

[B69] Othman A. A. Abou Rayia D. M. Ashour D. S. Saied E. M. Zineldeen D. H. El-Ebiary A. A. (2016). Atorvastatin and metformin administration modulates experimental *Trichinella spiralis* infection. Parasitol. Int. 65, 105–112. 10.1016/j.parint.2015.11.001 26546571

[B70] Pakoussi T. Mouzou A. P. Metowogo K. Aklikokou K. A. Gbeassor M. (2018). How do *Spondias mombin* L (*Anacardiaceae*) leaves extract increase uterine smooth muscle contractions to facilitate child birth in parturient women? Afr. Health Sci. 18, 235–243. 10.4314/ahs.v18i2.6 30602948 PMC6306990

[B71] Passaniti A. Taylor R. M. Pili R. Guo Y. Long P. V. Haney J. A.. (1992). A simple, quantitative method for assessing angiogenesis and antiangiogenic agents using reconstituted basement membrane, heparin, and fibroblast growth factor. Lab Invest. 67, 519–528. 1279270

[B72] Peng Q. Feng Z. Rui X. Zhixiong L. Mingcang C. Zhaolin S.. (2014). Identification of multiple constituents from seed of *Vaccaria segetalis* with an adsorbent-separation strategy based on liquid chromatography coupled to quadrupole time-of-flight mass spectrometry. Rapid Commun. Mass Spectrom. 28, 1243–1257. 10.1002/rcm.6893 24760565

[B73] Planas-Silva M. D. Waltz P. K. Kilker R. L. (2006). Estrogen induces death of tamoxifen-resistant MCF-7 cells: contrasting effect of the estrogen receptor downregulator fulvestrant. J. Steroid Biochem. Mol. Biol. 98, 193–198. 10.1016/j.jsbmb.2005.10.003 16464573

[B74] Qing L. I. Pan Z. L. Jie W. U. Zhang H. J. (2014). Study on extraction process and content determination of polysaccharides from *Semen vaccaria*. Sci. Technol. Food Indust. 35:299–299.

[B75] Qiu Y. Du B. Xie F. Cai W. Liu Y. Li Y.. (2016). Vaccarin attenuates high glucose-induced human EAâ? chy926 endothelial cell injury through inhibition of notch signaling. Mol. Med. Rep. 13, 2143–2150. 10.3892/mmr.2016.4801 26795539

[B76] Rong P. Zhu B. Li Y. Gao X. Ben H. Li Y.. (2011). Mechanism of acupuncture regulating visceral sensation and mobility. Front. Med. 5, 151–156. 10.1007/s11684-011-0129-7 21695619

[B77] Rosalía R. R. María Dolores H. Perona J. S. Valentina R. G. (2004). Potential vasorelaxant effects of oleanolic acid and erythrodiol, two triterpenoids contained in 'orujo' olive oil, on rat aorta. Br. J. Nutr. 92, 635–642. 10.1079/BJN20041231 15522132

[B78] Rostami A. Gamble H. R. Dupouy-Camet J. Khazan H. Bruschi F. (2017). Meat sources of infection for outbreaks of human trichinellosis. Food Microbiol. 64, 65–71. 10.1016/j.fm.2016.12.012 28213036

[B79] Sang S. Lao A. Chen Z. Uzawa J. And Y. F. (2003). “Chemistry and bioactivity of the seeds of *Vaccaria segetalis*,” in ACS Symposium Series Shanghai. 10.1021/bk-2003-0859.ch021

[B80] Sang S. Lao A. Wang H. Chen Z. Uzawa J. Fujimoto Y. (1999). Triterpenoid saponins from *Vaccaria segetalis*. J. Asian Nat. Prod. Res. 1, 199–205. 10.1080/10286029908039865 11254032

[B81] Sang S. M. Lao A. Leng Y. Gu Z. Chen Z. Uzawa J.. (2000d). Segetoside F a new triterpenoid saponin with inhibition of luteal cell from the seeds of *Vaccaria segetalis*. Tetrahed. Lett. 41, 9205–9207. 10.1016/S0040-4039(00)01710-X 12450258

[B82] Sang S. M. Lao A. N. Chen Z. L. Uzawa J. Fujimoto Y. (2000b). Three new triterpenoid saponins from the seeds of *Vaccaria segetalis*. J. Asian Nat. Prod. Res. 2, 187–193. 10.1080/10286020008039910 11256692

[B83] Sang S. M. Lao A. N. Leng Y. Cao L. Chen Z. L. Uzawa J.. (2002). A new triterpenoid saponin with inhibition of luteal cell from the seeds of *Vaccaria segetalis*. J. Asian Nat. Prod. Res. 4, 297–301. 10.1080/1028602021000049096 12450258

[B84] Sang S. M. Xia Z. H. Mao S. L. Lao A. Chen Z. L. (2000c). [Studies on the flavonol glycosides from the seeds of *Vaccaria segetalis*]. Zhongguo Zhong Yao Za Zhi 25, 221–222. 12512436

[B85] Sang S. M. Zou M. L. Lao A. N. Liang Z. Fujimoto Y. (2000a). A new triterpenoid saponin from the seeds of *Vaccaria segetalis*. ??????(???) 11, 49–52. 10.1021/cm990982a 11256692

[B86] Schieber M. Chandel N. S. (2014). ROS function in redox signaling and oxidative stress. Curr. Biol. 24, R453–462. 10.1016/j.cub.2014.03.034 24845678 PMC4055301

[B87] Shih C. C. Lin C. H. Lin W. L. (2009). Ameliorative effects of *Vaccaria segetalis* extract on osteopenia in ovariectomized rats. J. Nat. Med. 63, 386–392. 10.1007/s11418-009-0341-9 19475478

[B88] Shiva Kumar A. Jeyaprakash K. Chellappan D. R. Murugan R. (2017). Vasorelaxant and cardiovascular properties of the essential oil of Pogostemon elsholtzioides. J. Ethnopharmacol. 199, 86–90. 10.1016/j.jep.2017.01.036 28132862

[B89] Silva B. Guedes J. M. Arêde A. Costa A. (2012). Synthesis and bioactivity of secondary metabolites from marine sponges containing dibrominated indolic systems. Cheminform 17:6083. 10.3390/molecules17056083 22614862 PMC6268355

[B90] Sun H. Cai W. Wang X. Liu Y. Hou B. Zhu X.. (2017). Vaccaria hypaphorine alleviates lipopolysaccharide-induced inflammation via inactivation of NFkappaB and ERK pathways in Raw 264.7 cells. BMC Complement. Alternat. Med. 17:120. 10.1186/s12906-017-1635-1 28219355 PMC5319035

[B91] Sun J. Paddock C. Shubert J. Zhang H. B. Amin K. Newman P. J.. (2000). Contributions of the extracellular and cytoplasmic domains of platelet-endothelial cell adhesion molecule-1 (PECAM-1/CD31) in regulating cell-cell localization. J. Cell Sci. 113 (Pt. 8), 1459–1469. 10.1023/A:1005568132027 10725228

[B92] Tan N. H. Zhou J. (2006). Plant cyclopeptides. Chem. Rev. 106, 840–895. 10.1021/cr040699h 16522011

[B93] Tang X. Huang J. Xiong H. Zhang K. Chen C. Wei X.. (2016). Anti-Tumor Effects of the polysaccharide isolated from tarphochlamys affinis in H22 tumor-bearing mice. Cell. Physiol. Biochem. 39, 1040–1050. 10.1159/000447811 27537353

[B94] Tian-Yi L. (2011). Precious Mirror of Health. Shijiazhuang: China Medical Science Technology Press.

[B95] Tímár J. Döme B. Fazekas K. Janovics A. Paku S. (2001). Angiogenesis-dependent diseases and angiogenesis therapy. Pathol. Oncol. Res. 7, 85–94. 10.1007/BF03032573 11458270

[B96] Tong H. Gao X. Li Q. Liu J. Li N. Wan Z. (2011). Metabolic regulation of mammary gland epithelial cells of dairy cow by galactopoietic compound isolated from Vaccariae segetalis. Agric. Sci. China 10, 1106–1116. 10.1016/S1671-2927(11)60100-4

[B97] Tong H. Gao X. Sheng Z. Li Q. Li S. Li N.. (2013). Galactopoietic activity of dibutyl phthalate isolated from Vaccaria segetalis. J. Northeast Agric. Univ., 20, 28–33. 10.1016/S1006-8104(14)60043-X

[B98] Tuffrey M. Alexander F. Woods C. Taylor-Robinson D. (1992). Genetic susceptibility to chlamydial salpingitis and subsequent infertility in mice. J. Reprod. Fertil. 95, 31–38. 10.1530/jrf.0.0950031 1625247

[B99] Vandamme D. Landuyt B. Luyten W. Schoofs L. (2012). A comprehensive summary of LL-37, the factotum human cathelicidin peptide. Cell. Immunol. 280, 22–35. 10.1016/j.cellimm.2012.11.009 23246832

[B100] Viallard C. Larrivee B. (2017). Tumor angiogenesis and vascular normalization: alternative therapeutic targets. Angiogenesis 20, 409–426. 10.1007/s10456-017-9562-9 28660302

[B101] Vitagliano D. De Falco V. Tamburrino A. Coluzzi S. Troncone G. Chiappetta G.. (2010). The tyrosine kinase inhibitor ZD6474 blocks proliferation of RET mutant medullary thyroid carcinoma cells. Endocr. Relat. Cancer 18, 1–11. 10.1677/ERC-09-0292 20943719

[B102] Wang F. Chen S. Deng L. Chen L. Huang Y. Tian M.. (2019). Protective effects of astragaloside IV against LPS-induced endometritis in mice through inhibiting activation of the NF-kappaB, p38 and JNK signaling pathways. Molecules 24:373. 10.3390/molecules24020373 30669661 PMC6360020

[B103] Wang L. Cui D. Wang X. Zhang J. Yang Z. Qin Z.. (2015). Analgesic and anti-inflammatory effects of hydroalcoholic extract isolated from Semen vaccariae. Pak. J. Pharm. Sci. 28(3 Suppl), 1043–1048 26051722

[B104] Wang X. Dong H. Liu Y. Yang B. Wang X. Huang L. (2011). Application of high-speed counter-current chromatography for preparative separation of cyclic peptides from Vaccaria segetalis. J. Chromatogr. B 879, 811–814. 10.1016/j.jchromb.2011.02.001 21396894

[B105] Wei C. M. Su Y. J. Qin X. Ding J. X. Liu Q. Song F. M.. (2018). Monocrotaline suppresses RANKL-induced osteoclastogenesis *in vitro* and prevents LPS-induced bone loss *in vivo*. Cell. Physiol. Biochem. 48, 644–656. 10.1159/000491892 30025412

[B106] Xie F. Cai W. Liu Y. Li Y. Du B. Feng L.. (2015). Vaccarin attenuates the human EA.hy926 endothelial cell oxidative stress injury through inhibition of notch signaling. Int. J. Mol. Med. 35, 135–142. 10.3892/ijmm.2014.1977 25352009

[B107] Xu F. Hou B. Zhu X. Liu Y. Shi X. Li S.. (2019). Vaccaria n-butanol extract lower the production of proinflammatory cytokines and the infection risk of *T*. spiralis *in vivo. Acta Parasitol*. 64, 520–527. 10.2478/s11686-019-00064-6 31087260

[B108] Yu Y. Yuan X. Li P. Wang Y. Yu M. Gao X. (2020). Vaccarin promotes proliferation of and milk synthesis in bovine mammary epithelial cells through the Prl receptor-PI3K signaling pathway. Eur. J. Pharmacol. 880:173190. 10.1016/j.ejphar.2020.173190 32464193

[B109] Yun Y. S. Morita H. Takeya K. Itokawa H. (1997). Cyclic peptides from higher plants. 34. Segetalins G and H, structures and estrogen-like activity of cyclic pentapeptides from *Vaccaria segetalis*. J. Nat. Prod. 60, 216–218. 10.1021/np960617n 9157189

[B110] Yun Y. S. Shimizu K. Morita H. Takeya K. Itokawa H. Shirota O. (1998). Triterpenoid saponin from *Vaccaria segetalis*. Phytochemistry 47, 143–144. 10.1016/S0031-9422(97)00496-2 9429321

[B111] Zakaryan H. Arabyan E. Oo A. Zandi K. (2017). Flavonoids: promising natural compounds against viral infections. Arch. Virol. 162, 2539–2551. 10.1007/s00705-017-3417-y 28547385 PMC7087220

[B112] Zhang L. J. Zhu J. Y. Sun M. Y. Song Y. N. Rahman K. Peng C.. (2017). Anti-inflammatory effect of Man-Pen-Fang, a Chinese herbal compound, on chronic pelvic inflammation in rats. J. Ethnopharmacol. 208, 57–65. 10.1016/j.jep.2017.06.034 28652014

[B113] Zheng J. Ma L. T. Ren Q. Y. Li L. Zhang Y. Shi H. J.. (2014). The influence of astragalus polysaccharide and beta-elemene on LX-2 cell growth, apoptosis and activation. BMC Gastroenterol. 14:224. 10.1186/s12876-014-0224-8 25551689 PMC4297370

[B114] Zheng T. Chen W. Hu H. Wang Y. Harnett J. E. Ung C. O. L. (2020). The prevalence, perceptions and behaviors associated with traditional/complementary medicine use by breastfeeding women living in Macau: a cross-sectional survey study. BMC Complement. Med. Ther. 20:122. 10.1186/s12906-020-02921-8 32316951 PMC7175520

[B115] Zhi-hong Y. Cai-ying S. (1989). The effect of auricular plaster on gallbladder contraction. J. Gansu Coll. Trad. Chin. Med. 1, 31–32.

[B116] Zhou G. Tang L. Wang T. Zhou X. Wang Z. (2015). Phytochemistry and pharmacological activities of *Vaccaria hispanica* (miller) rauschert: a review. Phytochem. Rev. 15, 813–827. 10.1007/s11101-015-9425-1

[B117] Zhu-mo N. (2005). Materia Medica (Selected Chinese Ancient Books). Shanghai: Shanghai Science and Technology Press.

